# Advances in DNA Barcoding of Toxic Marine Organisms

**DOI:** 10.3390/ijms19102931

**Published:** 2018-09-26

**Authors:** Shaohua Gong, Yanfei Ding, Yi Wang, Guangze Jiang, Cheng Zhu

**Affiliations:** Key Laboratory of Marine, Food Quality and Hazard Controlling Technology of Zhejiang Province, College of Life Sciences, China Jiliang University, Hangzhou 310018, China; s1609071007@stu.cjlu.edu.cn (S.G.); s1509071024@stu.cjlu.edu.cn (Y.W.); s1709071008@stu.cjlu.edu.cn (G.J.)

**Keywords:** toxic algae, seafood safety, molecular identification, food safety

## Abstract

There are more than 200,000 marine species worldwide. These include many important economic species, such as large yellow croaker, ribbonfish, tuna, and salmon, but also many potentially toxic species, such as blue-green algae, diatoms, cnidarians, ctenophores, *Nassarius* spp., and pufferfish. However, some edible and toxic species may look similar, and the correct identification of marine species is thus a major issue. The failure of traditional classification methods in certain species has promoted the use of DNA barcoding, which uses short, standard DNA fragments to assist with species identification. In this review, we summarize recent advances in DNA barcoding of toxic marine species such as jellyfish and pufferfish, using genes including cytochrome oxidase I gene (*COI*), cytochrome b gene (*cytb*), *16S rDNA*, internal transcribed spacer (*ITS*), and Ribulose-1,5-bisphosphate carboxylase oxygenase gene (*rbcL*). We also discuss the application of this technique for improving the identification of marine species. The use of DNA barcoding can benefit the studies of biological diversity, biogeography, food safety, and the detection of both invasive and new species. However, the technique has limitations, particularly for the analysis of complex objects and the selection of standard DNA barcodes. The development of high-throughput methods may offer solutions to some of these issues.

## 1. Introduction

Toxic marine organisms are traditionally considered as those produce biotoxins or concentrate biotoxins from other organisms or the marine environment in their life periods. Toxic algae, such as dinoflagellates, diatoms and cyanobacteria, are associated with the production of many marine toxins, which can cause harmful algal blooms (HAB), paralytic shellfish poisoning (PSP), diarrhetic shellfish poisoning (DSP), neurotoxic shellfish poisoning (NSP), and ciguatera fish poisoning (CFP). Some seafood species can adapt to tolerate high levels of certain algal toxins. For example, softshell clams (*Mya arenaria*) from areas exposed to red tides can accumulate more PSP toxins and are more resistant to toxins, compared with sensitive clams from areas not exposed [1]. These kinds of toxic marine organisms are always highlighted in daily life, because toxins might lead to greater toxin resistance in seafood species and increased risk of toxins in humans, even resulting in long-term changes to communities and ecosystems.

Some marine organisms can still be health hazards, even though they do not produce or concentrate biotoxins in their life periods. Scombroid food poisoning is a foodborne illness resulting from eating spoiled (decayed) fish [2]. In fact, not only Scombroid fish, mackerel, tuna and bonito, but also fishes such as saira (*Cololabis saira*) and amberjack (Seriola) can cause food poisoning even after being cooked, due to the high content of histamine generated during the decay. These kinds of organisms are common but are not marked as toxic marine organisms. Because they can frequently cause food poisoning, these marine organisms are also potentially toxic and, therefore, discussed in the review.

Different organisms living in similar environments may become morphologically similar as a result of convergent evolution, while some marine organisms have diverse shapes at different developmental stages. These factors make it difficult to distinguish between toxic and non-toxic species by morphological methods alone. Some toxic species may be mixed with non-toxic species and processed into food products. Toxic marine algae, *Nassarius* spp., or pufferfish, for example, may be mislabeled and consumed by the public [3]. Toxic *Nassarius* spp. frequently causes poisoning incidents. From 1985 to 2000, 59 people in Ningbo, Zhejiang province in China were poisoned and 18 died due to *Nassarius* consumption [4]. Thus, it is very important to identify toxic marine species using reliable methods.

There are many ways to identify species, based on morphology, behavior, DNA, geography, and cross experiments. Unfortunately, most of these procedures do not work with processed marine food. Currently, there are three types of methods for identification of damaged or processed samples: morphological identification, protein identification, and DNA identification. Species identification of eggs and larvae can be difficult based on morphology alone [5], while tissue-specific proteins are often denatured during heating or processing, thus making protein identification unreliable [6]. Some species are highly polymorphic in color and markings even within a single population [7]. However, DNA analysis can identify species even from such samples as eggs, scales, fins, and processed food [8,9,10,11]. Thus, DNA-based identification is flexible and suitable for a wide range of situations.

DNA barcoding can be used to identify species rapidly by analyzing similarity of certain DNA fragments from samples with the sequences in a database, thus allowing identification and differentiation between species. Hebert et al. [3] first proposed the concept of DNA barcoding. They classified 200 different species from seven phyla and eight orders based on analysis of the cytochrome oxidase I gene (*COI*), with a minimum success rate of 96.4%. It was concluded that a classification system based on *COI* was generally applicable to all animals. A DNA barcode is currently defined by the Consortium for the Barcode of Life as a standard region of DNA that can be used to identify species efficiently. The main purpose of DNA barcoding is to identify unknown specimens [12]. With its high efficiency and adaptability mean, the technique of DNA barcoding has been widely used for the classification of marine species.

The increased use of DNA barcoding in various species has been accompanied by improvements in the technique with the ultimate aim to identify a universal DNA barcoding region that can be used to classify all species. However, due to the existence of pseudogenes, heteroplasmy, and different evolutionary rates, DNA barcoding should be used together with, rather than completely replace, conventional taxonomical methods [13,14].

**Figure 1:**
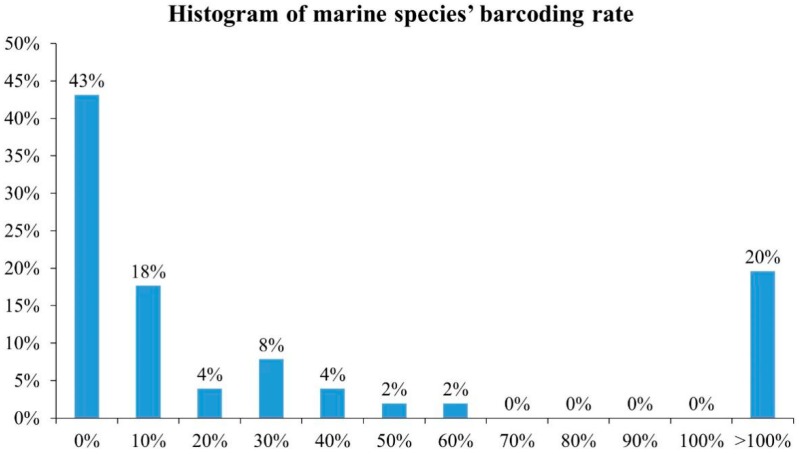
Histogram of marine species’ barcoding rates. The marine species’ barcoding rates were calculated using the data in Table 1. The percentages of the abscissa indicate the percentage of the number of species, and the percentage of the ordinate indicates the frequency of the barcoding rate.

**Figure 2:**
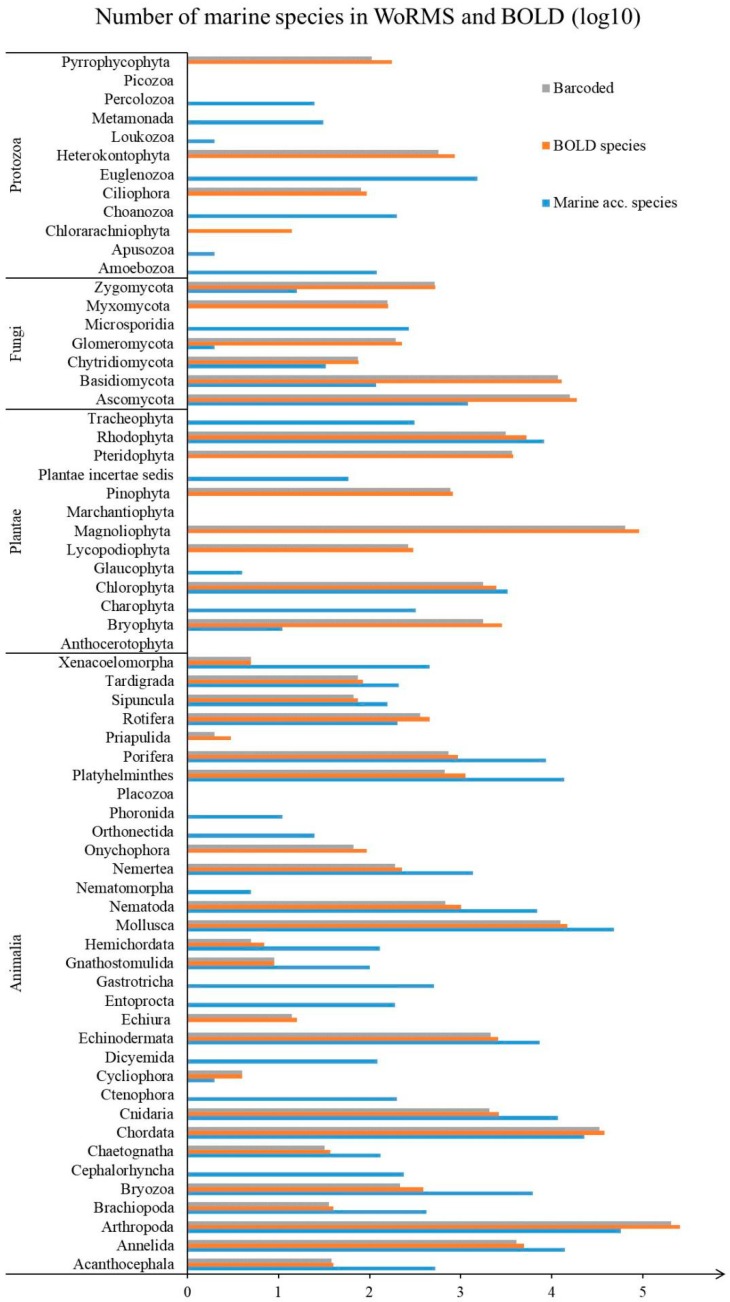
Number of marine species in WoRMS and BOLD. Due to the large difference among different phylum, all data are presented as logarithm of 10. Every unit in Figure 2 represents the difference of 10 times. Marine Acc.species represents the number of accepted marine species within the specific rank in WoRMS; BOLD species represents the number of species in BOLD; Barcoded represents the number of barcoded species in BOLD.

According to the World Register of Marine Species (WoRMS), http://www.marinespecies.org/), there were 240,659 accepted marine species worldwide as of February 2018. These comprised 201,195 species of Animalia, 12,129 species of Plantae, 21,075 species of Chromista, 2204 species of Protozoa, and 1673 species of Fungi. The Barcode of Life Data System (BOLD) is an informatics workbench to facilitate the acquisition, storage, analysis and publication of DNA barcode records [15]. The DNA barcoding rates according to statistics from the BOLD and WoRMS are shown in Table 1 and Figure 1, and the overall number of species and number of barcoded species in BOLD are shown in Figure 2. The abundant marine species worldwide include not only many of economic importance, but also many of toxic species. Here, we summarize recent research on DNA barcoding of toxic marine species to ensure human consumer protection and avoid food poisoning incident.

As the histogram shows, most (65%) marine species’ barcoding rate is less than 30%. Almost half (43%) of marine species’ barcoding rates fall in the range of 0–10%. The DNA barcoding rates are still relatively low. Meanwhile, 20% of marine species’ barcoding rates are larger than 100% because the data from BOLD are not focus on marine species.

## 2. Survey of Methodology

The data in this review were collected from notable databases. World Register of Marine Species (http://www.marinespecies.org/) is a common database that provides an authoritative and comprehensive list of marine organism names, including information on synonymy. Barcode of Life Data System (http://www.barcodinglife.org) is not only a database but also an analysis platform, which consists of data portal, registry of Brcode Index Numbers—BINs (putative species), data collection and analysis workbench. This review investigates basic papers, case studies and some frontier research.

## 3. DNA Barcoding Is widely Used in Toxic Marine Algae and Metazoans

A DNA barcode can be derived from the nucleus, mitochondrion and chloroplast. The most commonly used nuclear DNA barcodes are *18S rDNA*, *28S rDNA*, and internal transcribed spacer (*ITS*) [16,17,18]. Common mitochondrial DNA barcodes widely used in the animal kingdom include the genes for *COI*, cytochrome b gene (*cytb*), and control region (or displacement-loop, d-loop) [19] [20,21]. Common chloroplast DNA barcodes widely used in the plant kingdom include Maturase K gene (*matK*), Ribulose-1,5-bisphosphate carboxylase oxygenase gene (*rbcL*), and non-coding *trnH-psbA* spacer region [22,23,24]. Different DNA barcodes are suitable for different species, according to their specific sequence characteristics. This review focuses on the identification of several toxic marine species (diatoms, Cnidaria, Ctenophora, Mollusca, pufferfish, and tuna) and their commonly used DNA barcodes (Table 2, Table 3, Table 4, Table 5 and Table 6).

### 3.1. Toxic Marine Algae

Marine biotoxins are one kind of secondary metabolites produced by marine photosynthetic organisms including dinoflagellates, diatoms and cyanobacteria [44]. Under an appropriate environment, these microorganisms can grow and produce a large amount of biotoxins rapidly. Consequently, poisoning can occur when people swim, take bath or farm economic species in contaminated water. Furthermore, because of global climate change, eutrophication, urbanization and modern agriculture, the proliferation, frequency, and persistence of harmful algal blooms (HAB) are increasing in many parts of the world over the past decades [45].

Diatoms comprise one of the highest biomasses of phytoplankton in the ocean. They are widely distributed in both marine and fresh water, with an estimated 200,000 species [46]. *Pseudo-nitzschia* can be responsible for one of the most harmful algal blooms in coastal ecosystems, causing amnesic shellfish poisoning in humans as a result of the production of domoic acid [47].

Evans et al. [25] studied 34 diatom individuals from four genera and assessed the effectiveness of several genes (*COI*, *rbcL*, *18S rDNA* and *ITS*) for distinguishing cryptic species. They showed that the *COI* gene was best suited for DNA barcoding of most diatoms, with a greater divergence than *rbcL* and greater variability than *18S rDNA*. However, Moniz and Kaczmarska [27] found that *COI* had a low efficiency of amplification and that the *5.8S* + *ITS2* fragment had a higher success rate, making it a better candidate for diatom DNA barcoding.

Hamsher et al. [28] used *Sellaphora* isolates to test the ability of several markers (~1400 bp *rbcL*, 748 bp at the 3′ end of *rbcL*, LSU D2/D3, and UPA) to discriminate among closely related diatom species. The results suggested that *rbcL*-3P should be used as the primary marker for diatom DNA barcodes, as supported by Hamsher and Saunders [29]. However, after comparing several genes, MacGillivary and Kaczmarska [20] did not support the use of *rbcL* alone for DNA barcoding, and they recommended using *rbcL*-3P together with the *5.8S + ITS2* fragment, which has a higher mutation rate. Based on these studies, the *rbcL*-3P should be used as a primary marker and *5.8S + ITS2* fragment can also be used as a potential DNA barcodes due to its higher degree of variation.

### 3.2. Toxic Marine Invertebrates

#### 3.2.1. Mollusca

Harm of HAB is not limited to the organisms that generate HAB because their biotoxins can accumulate in other marine organisms (i.e., fish or shellfish) and end up in the human food web [48]. For example, some species of *Nassarius* are toxic at some growth seasons [49,50] and can pollute non-toxic marine species, such as softshell clams (*Mya arenaria*), an edible shellfish that is used in a variety of dishes. The claims from areas exposed to red tides are usually more tolerant to PSP toxins and can accumulate toxins at greater rates than those sensitive clams from unexposed areas [1]. The prevention of contaminated seafood reaching the markets is currently an effective way to ensure human health [51]. Legislative requirements are implemented to monitor shellfish to control the risk of shellfish poisoning to human consumers [52].

Zou et al. [30] showed that *COI* was better than *16S rDNA* when using distance-based methods for identifying 40 species of Neogastropoda. However, both genes showed 100% success rate in identifying all the samples when using the character-based method. In a different study, Zou et al. [31] analyzed different *Nassarius* species and found four cryptic species and one pair of synonyms using the *COI* gene. Couceiro et al. [17] used the *COI* gene to distinguish between the two morphologically similar species, *Nassarius nitidus* and *Nassarius reticulatus*, which showed a clear barcoding gap. Using *COI* and *28S rDNA*, Galindo et al. [32] analyzed the *Nassarius pauperus* complex from the eastern Indian Ocean and western Pacific Ocean, including a revised concept of *Nassa paupera* Gould, 1850, type species of the genus *Reticunassa* Iredale, 1936, and discovered six new species. Lobo et al. [53] pointed out that some of the so-called “universal primers” still failed to amplify *COI-5P* in some marine animal groups. Therefore, they designed a new pair of enhanced primers for the *COI-5P* region for a wide range of marine organisms, including Mollusca and Cnidaria. Thus, *COI* appears to be the most widely used DNA barcoding gene in Mollusca.

#### 3.2.2. Cnidaria

In addition to the shellfish mentioned above, there are other toxic marine invertebrates including jellyfish, which can cause skin damage to human, one of the most common poisoning incidents for treatment in clinics and hospitals in highly industrialized countries [54]. The phylum Cnidaria consists of four categories including Anthozoa, Cubozoa, Hydrozoa and Scyphozoa, the last three of which are collectively known as Medusozoa. Jellyfish use compounds with neurotoxic and cardiotoxic effects for hunting and for defense against predators or other potential threats [55]. Jellyfish toxins cause toxic effects in diverse organisms and can trigger local and systemic reactions [56].

Since Cnidaria shows far lower *COI* divergences than any other phylum, efforts have been taken to find better DNA barcodes for Cnidaria. Moura et al. [33] studied 56 sequences from the Hydrozoan families Eudendriidae, Lafoeidae, Haleciidae, Sertulariidae, Plumulariidae, and Aglaopheniidae, and found that *16S rDNA* was a useful DNA barcoding tool for Hydrozoa. Armani et al. [34] used five sets of primers for the *COI* gene to identify jellyfish products and found that 100% of ready-to-eat jellyfish products were mislabeled. McInnes et al. [35] used *18S rDNA* to study the occurrence of jellyfish predation by black-browed and Campbell albatross. McFadden et al. [57] reviewed the limitation of mitochondrial DNA barcoding in Octocorallia. Their recent studies revised Genus *Ovabunda* in the Red Sea and divided four clades using three mitochondrial (*mtMutS*, *COI*, and *ND2*) and four nuclear (*ITS*, *28S rDNA*, *ATPS*α, and *ATPS*β) genes [36]. Miranda et al. [37] conducted a comprehensive molecular phylogenetic analysis of Staurozoa with a set of DNA barcodes (mitochondrial markers *COI* and *16S rDNA*, and nuclear markers *ITS*, *18S rDNA* and *28S rDNA*) using three methods (Parsimony phylogenetic hypothesis, Maximum likelihood phylogenetic hypothesis and Bayesian phylogenetic hypothesis). The comprehensive comparison of these five DNA barcodes showed that only *28S rDNA* supported each main group observed in the three methods. Therefore, nuclear genes (*ITS*, *18S rDNA* and *28S rDNA*) and their combination with mitochondrial genes are increasingly used for DNA barcoding in Cnidaria.

### 3.3. Toxic Marine Fish

Fishes are the largest and most diverse vertebrates, with the value of many commercial fisheries exceeding US$ 200 billion [5]. Pufferfishes are well-known to be toxic and are thus the subject of a unique type of commercial fishery. The high rate of mislabeling of marine fish in markets makes it highly urgent to review the research and development of toxic commercial fisheries [34].

#### 3.3.1. Pufferfish

Most members of the family Tetraodontidae carry tetrodotoxin (TTX), which is typically concentrated in the liver, but also in the ovaries, intestines, and skin. Tetrodotoxin is a heat-stable neurotoxin that can cause weakness or paralysis and death [58]. There were 28 cases of pufferfish poisoning in Florida, New Jersey, Virginia, and New York from January 2002 to May 2004 [59].

Amaral et al. [34] used the *COI* gene to analyze the pufferfish in Tocantins River and identified a new species of the genus *Colomesus*, which was formerly thought to contain only two species, *C. asellus* and *C. psittacus*. Huang et al. [38] used the *cytb* gene as the basis for identifying pufferfish. This system was tested in several specimens of pufferfish, as well as in simulated products and commercial samples. They reported that 3.92% of the tested samples were from toxic species using the *cytb* gene as a barcoding marker. A reported study used Full and mini *COI* DNA barcodes to identify 68 ethnic seafood products from an Italian market [39] and showed that two poisonous pufferfish samples forbidden in the European Union were wrongly labelled as squid. Tuney [40] used *16S rDNA* and *cytb* to identify *Lagocephalus sceleratus* and *Lagocephalus spadiceus*, which are well-known invasive marine species from the Red Sea, and concluded that *cytb* was more useful than *16S rDNA* in this study. Thus, *COI* and *cytb* are the most accepted DNA barcodes in Pufferfish.

#### 3.3.2. Scombridae

Although studies of toxic marine fishes often focus on fishes that produce and accumulate specific toxins, other fishes, such as the genus *Scombermorus* and tuna, do not contain toxins but do have high levels of vitamins and histamine that can also cause harmful effects, even though they are often not considered as poisoning incidents. Scombridae fishes, which contain 15 genera and 51 species, have a worldwide importance for their economic and ecological value [16].

Vinas and Tudela [41] found that the mitochondria control region or displacement-loop (d-loop) combined with *ITS1* could fully distinguish the eight *Thunnus* species in different kinds of processed tissue. Pedrosa-Gerasmio et al. [42] and Kumar et al. [19] used only d-loop to identified Scombridae fish. Using *COI* in larval fishes, Seyhan and Turan [43] studied nine Scombrid species in Turkey and authenticated the efficacy of *COI* in identifying the Scombrid species with designated barcodes. *COI* and d-loop are the common DNA barcodes for identifying Scombridae fish.

## 4. Disadvantages of DNA Barcoding

Species identification using DNA barcoding-based methods have advantages over conventional morphological methods, but they also have limitations. A major issue with DNA barcoding from the very beginning of its application is the failure to find the universal primer or universal DNA barcode for a specific target organism. The evolution rates of nuclear and mitochondrial DNA are different. *COI* may be useful to some species, but the evolution rates of mitochondrial DNA are not uniform for all species’ evolution. The same is true for the nuclear DNA barcodes. Furthermore, pseudogenes and heteroplasmy can complicate the DNA barcoding research. Pseudogenes can lead to false division of one species into several species by mistake [60]. Pseudogenes can cause heteroplasmy, resulting in the coexistence of more than one type of mtDNA in the same individual, which can significantly decrease the reliability of species identification by DNA barcoding and increase the complexity of the database [61]. Differences in evolutionary rates provide various DNA barcoding options but make it difficult to find a universal DNA barcode for all species. Most studies using DNA barcoding use Sanger sequencing, which determines the sequence of only one sample. If DNA samples contain more than one template, the determined sequence will produce misleading results. Pseudogenes can have similar problems [62]. Batovska et al. [63] reported that Sanger sequencing is not an appropriate method for characterizing *ITS2* in the majority of mosquito species for the variety of polymorphisms in the gene.

## 5. Fluorescence Methods of DNA Barcoding

### 5.1. Real-Time Fluorescence PCR

Unlike traditional PCR method, real-time fluorescence PCR (RT-PCR, qPCR) allows rapid determination of target gene in samples with higher sensitivity. By using the fluorescent signal, the PCR procedure also has an advantage for monitoring target sequences with very low DNA concentrations. Smith et al. [64] developed qPCR assays to determine the presence of *Gambierdiscus/Fukuyoa* species in environmental samples, which contains potentially toxic species. Farrell et al. [8] used DNA barcodes *ITS*, *5.8S rDNA* and the *sxtA* gene specific to the saxitoxin synthesis pathway of *Alexandrium minutum* to detect saxitoxin-producing microalgae in shellfish. Both kinds of genes showed reliable results. Mullet roe is commonly adulterated by the addition of other species, such as escolar and oilfish. Kuo et al. [65] developed real-time PCR methods that could be used to verify the labelling of actual mullet roe products. Further tests on a random survey of commercial fish roe products demonstrated the efficacy of the technique in the detection of mullet DNA. Water, seabream *Pagrus auratus* and seabass *Dicentrarchus labrax* samples were collected from Abu Qir, Alexandria to evaluate the concentrations of dioxin. RT-PCR assays were conducted to verify the expression of certain immune genes in the fish species resulting from water pollution [66]. DNA barcoding has also been used to assess the genomic identity of the microalga species *Scenedesmus* sp. Barcode markers *rbcL* and *ITS*1-5.8S*-ITS*2 were sequenced and the obtained genomic information was used to design a quantitative PCR assay to precisely quantify the *S. almeriensis* concentrations in microalgal cultures of industrial interest [67]. Park et al. [68] used *COI* gene as DNA barcode to identify 14 species of microalgae from the South Sea of Korea, and found that species-specific PCR of the *COI* gene could be used to monitor the seasonal dynamics of microalgae in the South Sea of Korea.

### 5.2. High Resolution Melting

DNA barcoding, usually mini-barcoding, can be combined with high resolution melting (HRM) for the authentication of many commercial species from fake products such as Gadidae fish [69], Monofloral honeys [70] as well as herbal medicines from toxic species, such as *Crotalaria spectabilis* Roth. in *Thunbergia laurifolia* Lindl. [71], *Armeniacae semen* amarum in *Persicae semen* [72].

## 6. High-Throughput Methods of DNA Barcoding

### 6.1. DNA Metabarcoding

As discussed above, many toxic species are associated with toxic algae and biotoxins. Marine biotoxins regularly occur along the coast, with serious consequences for the environment as well as the food industry. Monitoring of these compounds in seawater is important to assure the safety of human consumers. However, early determination of marine biotoxins in seawater to prevent seafood contamination events has not been explored [44]. By using high-throughput methods of DNA barcoding, it is feasible to develop a reliable taxonomic identification tool. The development of metabarcoding approaches was aided by the advancement of next-generation sequencing (NGS) [73]. DNA metabarcoding is a high-throughput method of taxon identification based on very short (usually <100 bp) but informative DNA fragments [74]. In this respect, metabarcoding differs from normal DNA barcoding, because classic DNA barcoding aims to identify complete genomic DNA up to species level, and metabarcoding aims to identify degraded DNA samples (eDNA) up to the family or higher levels.

Lallias et al. [75] investigated the richness of marine nematode species by high-throughput sequencing using 18S as a DNA barcode. The abundances of certain species can be used to investigate hunters’ diets. Salvitti et al. [76] used DNA metabarcoding to conduct diet analysis to verify the source of TTX. Certain applications, such as the identification of gastric contents, present additional problems. Gastric contents can be analyzed by DNA barcoding for drugs and poisons [77], but can be very complex depending on the species, feeding habits, and environment. A high-throughput method would be better for analysis of such complex systems. Furthermore, DNA metabarcoding can be used in the identification of amphibians and bony fish. Evans et al. [78] conducted a DNA metabarcoding analysis using environmental DNA (eDNA) sampling to measure species diversity in aquatic. Similarly, Valentini et al. [79] used eDNA barcoding extracted from water samples to explore the rich of amphibians and bony fish. They argued that the DNA metabarcoding has the potential to become the next-generation tool for ecological studies and biodiversity monitoring in aquatic ecosystems.

### 6.2. Microarray

Microarray analysis represents another high-throughput method for identifying species. Microarray, also known as gene chip or biochip technology, can be used to analyze a large number of genes simultaneously. DNA barcodes can be used to design probe sequences in microarray analysis. Kochzius et al. [65] used *COI* probes to identify 30 fish species. Therefore, microarray-based identification methods will play a larger role in molecular species identification in the near future, especially for complex mixtures [80]. Increased use of DNA chips will help develop new methods for DNA barcoding [81].

## 7. Other Methods Used with DNA Barcoding

Besides the methods mentioned above, there are other techniques that can be combined with DNA barcoding. DNA barcoding can combine with nanotechnology. NanoTracer developed by Valentini et al. [82] simplified the analytical steps with standard DNA barcoding analysis and making it sequencing-free and portable outside specialized laboratories. The design of the specifically labeled primers involves their linkage through an antigen-antibody reaction to gold nanoparticles. Taboada et al. [83] developed species-specific lateral flow dipstick (LFD) assays for species identification in food products, in which gold nanoparticles enabled visual detection with good sensitivity even for processed samples. In addition, nanobiosensors can achieve on site, in situ and online measurements, and exhibit an unprecedented level of performance and the ability to “nano-tune” various properties to achieve the desired levels of sensitivity and detection limit. Their applications include a barcode assay for genetically modified organisms (GMO) using Surface Enhanced Raman Spectroscopy (SERS), and a mobile barcode enzymatic assay [84].

The loop-mediated isothermal amplification (LAMP) method is a highly sensitive method based on the use of a set of four specially designed primers that recognize a total of six distinct sequences of the target DNA [85]. LAMP has been used in detection of toxic and non-toxic species [86,87,88]. Su et al. [89] successfully developed the one-step RT-LAMP technique, which is a rapid and reliable method to detect HuNoV in stool samples and oysters with high sensitivity. Furthermore, LAMP methods have already been successfully developed for the detection of foodborne bacteria and fungi [85,90].

Digital PCR (dPCR) is a method that allows for absolute quantitation of nucleic acids, which has been widely used in cancer mutation studies, environmental monitoring, low-level pathogens and rare genetic sequences. dPCR is more sensitive than traditional qPCR and has been used to estimate eDNA concentration, fish abundance and biomass [91]. Multiplex digital PCR was used to co-amplify 16S *rDNA* and a metabolic gene from single bacterial cells [92,93]. *Singapore grouper iridovirus* (SGIV) is one of the major causative agents of fish diseases and has caused significant economic losses in the aquaculture industry. Droplet digital PCR (ddPCR) confirmed ribavirin, harringtonine, and 2-hydroxytetradecanoic acid (2-HOM) were effective at inhibiting SGIV infection [94]. In addition, ddPCR was also considered more suitable for the detection of *Z. marina* DNA from marine sediments [95].

## 8. Summary and Conclusions

Through the rapid development over the past 15 years or so, DNA barcoding has represented a well-proven molecular tool on taxonomic research. It relies on sequence variation within a short and standardized region of the genome to provide accurate species identification [13]. DNA barcoding lends aspiration to the assessment of biodiversity in a more accurate as well as inexpensive manner. Several DNA barcodes including mitochondrial *COI* gene, *rbcL*, *matK*, *trnH-psbA*, and *ITS* (nuclear internal transcribed spacer) have been extensively used as a global bio-identification system for detecting the alien species that invade different ecosystems [4]. Currently, the mitochondrial genes coding *COI* and *cytb* are considered reliable DNA barcodes for the identification of toxic marine species and seafood products [34,38,39,69]. Species adulteration is common for China’s roasted Xue Yu fillet products. Xiong et al. [96] applied DNA and mini-DNA barcoding for the species identification of 153 roasted Xue Yu fillet products from 30 brands. Giusti et al. [97] selected *cytb* gene as the molecular target to identify sixteen mislabeled commercial products containing pufferfish with degraded DNA. *Cytb* dataset’s phylogenetic analysis supported the most recent species classification of the *Lagocephalus* genus and highlighted the presence of toxic *L. spadiceus* in the products.

However, DNA barcoding has limitations. For example, species-specific universal primers or universal DNA barcodes are hard to find. Differences in evolutionary rates provide various DNA barcoding options but make it difficult to find a universal DNA barcode for all species [98,99]. Therefore, techniques of DNA barcoding for species identification are rapidly evolving as well. Traditional methods such as PCR-RFLP, PCR-SSCP and species-specific PCR are reliable but cannot meet the demands for high throughput, high speed, high sensitivity, standardization and automation. Fluorescence methods and high-throughput methods of DNA barcoding show high potential for characterizing samples to species-level. New molecular techniques such as LAMP and dPCR can be combined with DNA barcoding with high sensitivity and high speed. All these methods shall play an important role in species identification, specimen identification, biodiversity investigation, HAB forecast, detection of pathogens and seafood spoilage, and assessment of food authenticity. In particular, the use of molecular authentication methods has become one of the prospective standards to ensure food safety in the future [100].

In view of the current development, Sanger sequencing methods and fluorescence methods are rapid, cost saving but low throughput while high-throughput methods are suitable for large scale, and complex systems but are expensive and require long turn-around time. With the development of sequencing technologies, MinION-based DNA barcoding methods has been used in biodiversity research which are cost-effective and portable [35,101,102]. Furthermore, nanosensors can achieve on site, in situ and online measurements, and exhibit an unprecedented level of performance and the ability to “nano-tune” various properties to achieve the desired levels of sensitivity and detection limit. Nanobiosensors are used for the monitoring of food additives, toxins and mycotoxins, microbial contamination, food allergens, nutritional constituents, pesticides, environmental parameters, plant diseases, and genetically modified organisms [84]. We anticipate that nanotechnology will be widely used in conjunction with DNA barcodes, and standardized and automated high-throughput methods will become the mainstream of DNA barcode research in the future.

## Figures and Tables

**Table 1:** Table of accepted species, barcoded species, barcoding rate in BOLD and WoRMS.

Kingdom	Phylum	Acc.species	Barcoded	Rate
**Animalia**	Acanthocephala	529	38	7%
	Annelida	13,949	4055	29%
	Arthropoda	57,340	202,937	354%
	Brachiopoda	421	36	9%
	Bryozoa	6147	216	4%
	Cephalorhyncha	236	0	0%
	Chaetognatha	131	32	24%
	Chordata	22,891	33,226	145%
	Cnidaria	11,719	2046	17%
	Ctenophora	200	0	0%
	Cycliophora	2	4	200%
	Dicyemida	122	0	0%
	Echinodermata	7336	2137	29%
	Entoprocta	190	0	0%
	Gastrotricha	506	0	0%
	Gnathostomulida	101	9	9%
	Hemichordata	130	5	4%
	Mollusca	47,673	12,458	26%
	Nematoda	6897	680	10%
	Nematomorpha	5	0	0%
	Nemertea	1363	191	14%
	Orthonectida	25	0	0%
	Phoronida	11	0	0%
	Placozoa	1	0	0%
	Platyhelminthes	13,596	663	5%
	Porifera	8653	731	8%
	Rotifera	201	360	179%
	Sipuncula	156	67	43%
	Tardigrada	209	75	36%
	Xenacoelomorpha	454	5	1%
**Plantae**	Bryophyta	11	1754	15,945%
	Charophyta	322	0	0%
	Chlorophyta	3247	1764	54%
	Glaucophyta	4	0	0%
	Plantae incertae sedis	59	0	0%
	Rhodophyta	8173	3135	38%
	Tracheophyta	313	0	0%
**Fungi**	Ascomycota	1202	15,779	1313%
	Basidiomycota	118	11,725	9936%
	Chytridiomycota	33	75	227%
	Glomeromycota	2	193	9650%
	Microsporidia	270	0	0%
	Zygomycota	16	515	3219%
**Protozoa**	Amoebozoa	120	0	0%
	Apusozoa	2	0	0%
	Choanozoa	198	0	0%
	Euglenozoa	1528	0	0%
	Loukozoa	2	0	0%
	Metamonada	31	0	0%
	Percolozoa	25	0	0%
	Picozoa	1	0	0%

Acc.species represents the number of accepted marine species within the specific rank in World Register of Marine Species (WoRMS); Barcoded represents the number of barcoded species in Barcode of Life Data System (BOLD); Rate represents the barcoding rate that calculated by divide Barcoded by Acc.species.

**Table 2:** Commonly used DNA barcodes in Diatom.

Diatom	DNA Barcodes
Recommended DNA Barcodes	*rbcL*-3P *5.8S* + *ITS2* fragment
Genus *Sellaphora*	*COI* [25]
Genus *Pinnularia*	
Genus *Eunotia*	
Genus *Tabularia*	
Class Mediophyceae	*5.8S + ITS2* fragment [26]
Class Bacillariophyceae	
Genus *Coscinodiscus*	*5.8S + ITS2* fragment [27]
Genus *Melosira*	
Genus *Minutocellulus*	
Genus *Chaetoceros*	
Genus *Eunotia*	
Genus *Nitzschia*	
Genus *Pseudonitzschia*	
Genus *Sellaphora*	*rbcL*-3P [28,29]
Class Mediophyceae	*rbcL*-3P with *5.8S + ITS2* fragment [4]
Class Bacillariophyceae	

**Table 3:** Commonly used DNA barcodes in Mollusca.

Mollusca	DNA Barcodes
Recommended DNA Barcodes	*COI*
Order Neogastropoda	*COI* [30,31]
Genus *Nassarius*	*COI* [31]
*Nassarius nitidus* *Nassarius reticulatus*	*COI* [17]
Genus *Reticunassa*	*COI* and *28S rDNA* [32]

**Table 4:** Commonly used DNA barcodes in Cnidaria.

Cnidaria	DNA Barcodes
Recommended DNA Barcodes	Nuclear DNA barcoding (*ITS*, *18S rDNA*, and *28S rDNA*)
Family Eudendriidae	*16S rRNA* [33]
Family Lafoeidae	
Family Haleciidae	
Family Sertulariidae	
Family Plumulariidae	
Family Aglaopheniidae	
Family Catostylidae	*COI* [34]
Family Cassiopeidae	
Family Cepheidae	
Family Lychnorhizidae	
Family Rhizostomatidae	
Family Cyaneidae	
Family Pelagiidae	
Family Ulmaridae	
Class Scyphozoa	*18S rDNA* [35]
Genus *Ovabunda*	*mtMutS, COI, ND2* and *ITS*, *28S rDNA*, *ATPS*α, *ATPS*β [36]
Class Staurozoa	*COI*, *16S rDNA* and *ITS*, *18S rDNA*, *28S rDNA* [37]

**Table 5:** Commonly used DNA barcodes in Pufferfish.

Pufferfish	DNA Barcodes
Recommended DNA Barcodes	*COI*, *cytb*
Family Triodontidae	*COI* [34]
Family Diodontidae	
Family Tetraodontidae	
Genus *Takifugu*	*cytb* [38]
Genus *Lagocephalus*	
Genus *Sphoeroides*	
*Lagocephalus* spp.	Full and mini *COI* [39]
*Lagocephalus sceleratus*	*cytb* [40]
*Lagocephalus spadiceus*	

**Table 6:** Commonly used DNA barcodes in Scombridae.

Scombridae	DNA Barcodes
Recommended DNA Barcodes	*COI*, d-loop
Genus *Thunnus*	d-loop *+ ITS1* [41]
*Thunnus albacares*	d-loop [42]
*Thunnus obesus*	
*Auxis thazard*	d-loop [19]
*Euthynnus affinis*	
*Katsuwonus pelamis*	
*Thunnus tonggol*	
*Thunnus albacares*	
*Thunnus alalonga*	*COI* [43]
*Thunnus thynnus*	
*Euthynnus alletteratus*	
*Auxis rochei*	
*Katsuwonus pelamis*	
*Sarda sarda*	
*Scomber colias*	
*Scomber scombrus*	
*Scomberomorus commerson*	
